# Considering late effects costs in radiotherapy funding

**DOI:** 10.1093/bjr/tqaf254

**Published:** 2025-10-21

**Authors:** Daniel Hutton, Imogen Powell Brown, Nicola Thorp, James Thomson, Ran MacKay, Liesl Hacker, Lisa Ashmore, John Hayes, John Archer, Carl Rowbottom

**Affiliations:** NW Radiotherapy Specialised Services Clinical Network, Manchester M20 4BX, United Kingdom; Lancaster University, Lancaster Medical School, Lancaster LA1 4AT, United Kingdom; NW Radiotherapy Specialised Services Clinical Network, Manchester M20 4BX, United Kingdom; The Christie, Radiotherapy, Manchester M20 4Bx, United Kingdom; The Clatterbridge Cancer Centre, Radiotherapy, Liverpool L7 8YA, United Kingdom; The Christie, Radiotherapy, Manchester M20 4Bx, United Kingdom; The Christie, Radiotherapy, Manchester M20 4Bx, United Kingdom; Lancaster University, Lancaster Medical School, Lancaster LA1 4AT, United Kingdom; Cheshire and Merseyside Cancer Alliance, The Clatterbridge Cancer Centre, Liverpool L7 8YA, United Kingdom; The Christie, Radiotherapy, Manchester M20 4Bx, United Kingdom; The Clatterbridge Cancer Centre, Radiotherapy, Liverpool L7 8YA, United Kingdom

**Keywords:** radiotherapy, late effects, living with and beyond cancer, cancer side effects, PROMs, PREMs, supportive oncology

## Abstract

Radiotherapy treatment can have transformative effects on a patient’s overall health and wellbeing, yet current funding models are constrained to curative and palliative aspects of treatment delivery. This therapeutic focus, obscures wider costs associated with radiotherapy, both at a service level and for individual patients and their families. It is essential that policy and services consider quality of life after treatment, including identification and management of long-term side effects. Currently, a lack of service provision means that many patients have no access to services equipped to manage late toxicity or are utilising inappropriate services for their needs which could also be more costly for commissioners. As Integrated Care Boards (ICBs) take greater responsibility for the whole cancer pathway there are potential patient and cost benefits of rolling out more supportive oncology and late effects services. This should be supported with better data, including Patient Reported Data (PRD) and research on the level of need for broader aspects of radiotherapy and post treatment aspects of patient experience.

This paper is part of a series of three papers, on (1) radiotherapy tariff, (2) radiotherapy capital spending and (3) holistic aspects of radiotherapy funding, which together consider what a sustainable, innovative and person centred radiotherapy funding model looks like as specialised services are delegated to Integrated Care Boards.

## Background

Radiotherapy is a highly effective treatment for cancer with over 100 000 patients in England receiving it each year.^1^ Radiotherapy uses highly targeted beams of radiation to treat tumours effectively, but in doing so can also affect nearby healthy cells. While decades of research have focussed on making treatment more precise and personalised to individual patients, radiotherapy can still cause long term and debilitating psychological and physical side effects.^2^

These can include fatigue, neurocognitive impacts, changes in physical capabilities, sexual and fertility problems, alongside psychological impacts such as fear of recurrence, depression, and anxiety.^2^ It is estimated that the quality of life for around a third of cancer survivors is negatively impacted by long-term consequences of cancer or its treatment.^2^ Advances in the diagnosis and treatment of cancer has positively impacted survival, however, this also means that the number of people living with long term impacts of cancer is growing.^3^

## Late effects services

Specialist late effect and supportive oncology services are able to respond to more people living after cancer by providing a specialist multidisciplinary package of care to patients experiencing late effects.^4^ Specialist services can provide management of physical and psychological symptoms and side effects to improve rehabilitation, secondary cancer prevention and support quality of life through survivorship.^5^

NHS England expects radiotherapy service providers to support patients with the management of late effects, including with the provision of specialist services.^6^ However, funding for care providers to respond to these side effects is omitted in current reimbursement arrangements for radiotherapy services. The current funding system, which was initially introduced over 10 years ago, only sets out funding for the technical aspects of radiotherapy planning and treatment, neglecting wider elements of the impacts of radiotherapy on patients including late effects and supportive oncology services.

Without a national model for how side effects are assessed or managed, specialist post-cancer treatment is inconsistent across the NHS and often reliant on charity funding. Many patients will depend on non- specialist services, such as general practice, where healthcare practitioners may have limited awareness of treatment side effects^4^^,^^7^ or utilise emergency care.^8^^,^^9^ Patient survey results indicate a growing demand for better post-treatment care and support to manage the long-term or delayed effects of cancer treatment.^10^

Specialist late effect and supportive oncology services could have important cost benefits for commissioners with evidence showing that funding supportive oncology services can be a more effective use of healthcare resources, by reducing the number of patients presenting to emergency services and by supporting secondary cancer diagnoses.^8^^,^^9^ A supportive oncology service in Royal Sussex County Hospital, Brighton brought together a multidisciplinary team to identify cancer patients within the acute setting to determine need and to provide links to oncological expertise where indicated.^11^ A review of the service found that it reduced emergency hospital admissions by an average 0.95 admissions per patient and length of stay by an average of 1.43 days. The costs of the service were returned with a benefit cost ratio of 1.4.^11^

The delegation of certain specialised services to Integrated Care Boards (ICBs) will give ICBs responsibility for commissioning across the entire cancer pathway. The reform was motivated in part by aims to create a more preventative health care system, considering that a greater focus on upstream intervention could reduce demand and cost for certain specialised services.^12^ However, the reforms also give the Government and NHS an opportunity to restructure cancer service funding to reflect holistic outcomes of treatment. Whole pathway commissioning enables ICBs to predict service demand across their local systems, supporting patients with late effects to be identified and managed by appropriate services. This approach could introduce important incentives ICBs to pursue more cost-effective models of care for these patients.

## Patient reported data

Whilst it is essential that patient demand for late effects care is met within current understanding of patient need, better data on the level of need for these aspects of patient experience is needed for a comprehensive and long-term policy response. Data used to inform decisions in radiotherapy practice and policy are often limited to medicalised and economic measures. This technical focus of radiotherapy has often silenced patient voices, with a result that policy and practice reflects advanced treatments options but perhaps neglects more holistic elements.^13^ A wider, holistic appreciation should incorporate other data sources, including on late effects from treatment, to inform priorities, policy, and practice.^14^

Patient Reported Data (PRD) comprising of Patient Reported Outcome Measures (PROMs) and Patient Reported Experience Measures (PREMs) are valuable tools to understand people’s experiences and the longer term and broader effects of radiotherapy. However, the monitoring of patient’s broader experience using these tools to determine effects of radiotherapy is inconsistent and incomplete in England.

Patient experience can be captured through surveys, with oncology having two large examples. The National Cancer Patient Experience Survey (NCPES) and The National Inpatient survey, while valuable, have limitations from a radiotherapy perspective and include only two questions relating to radiotherapy.^15^

Patient Reported Outcome Measures (PROMs) refer to the information provided by a patient regarding their own health using a self-reporting medium. PROMs collect patient’s perspectives of health, illness and the effects of health interventions, commonly completed through questionnaires. PROMs use improves patient-centred care and quality of life through providing clinicians with tailored, actionable patient data. Disease specific PROMs offer an additional lens to assess the impacts of treatment, working as a valuable tool to amplify the patient experience of late effects and ensure that interactions within radiotherapy services and survivorship care are guided by patient’s priorities.^16–18^

The radiotherapy service specification states that all radiotherapy services should routinely collect and analyse clinical outcome data, including PROMs.^6^ However, despite the support for PROMs, a recent review of practice in the UK reported less than a quarter of respondents indicated that PROMs were employed as standard of care in their radiotherapy department.^17^ Research shows that funding, infrastructure, such adequate information technology, and the time to integrate data collection within clinical workflows are barriers to the routine collection of PROMs.^19–21^ Reviews of the use of PROMs within radiotherapy have also highlighted that because the measure is not specific to radiotherapy it does not contain the specificity to capture all impacts of treatment.^22^^,^^23^

The UK Government’s 10 Year Health Plan indicates that PRD will become important for determining new health quality metrics as part ambitions to make the health system more transparent and responsive for patients.^24^ Comprehensive collection of PRD is needed within radiotherapy to build valuable data on patient experience and estimate the level of need. A routine national patient experience survey for radiotherapy would serve as a valuable tool for commissioners and service leads to design and fund services that align with patient needs and expectations.

Meanwhile, the expectation on radiotherapy providers to routinely collect PROMs data should be strengthened. Research has suggested that a potential solution for this within radiotherapy could come from reimbursing providers for collecting PROMs data through their radiotherapy tariff payment, reflecting that funding and infrastructure is a barrier to collection.^16^^,^^17^ This would financially incentivise the collection of PROMs and in turn build valuable patient experience data. Developing PROMs to cover the broad range of side effects related to radiotherapy and cancer is needed for this to be most effective. Finally, listening directly to patient experiences through ‘patient narratives’ exercises could give valuable qualitative insight into care provision.^14^

## Conclusion

Funding for cancer providers should encompass broader aspects of patient experience and interactions with care. Late effects from cancer and cancer treatment, including radiotherapy, are well understood, and while decades of research have sought to make treatment kinder and more personalised, patients expect greater provision of post cancer treatment support.^11^

The upcoming National Cancer Plan provides an opportunity to determine what funded, holistic care for people living with and beyond cancer looks like. This could consider a national model on late effects service provision, which can be contextualised to local settings and adapted for patients with various tumour sites. Building on evidence from specialist services already in place across the country can support this. To implement this, the Government should direct and fund ICBs to commission late effects or supportive oncology services nationally. Appropriate funding of late effects services by ICBs could help ensure that patients receive high-quality care from the most suitable providers. As the number of people living with long-term effects of cancer continues to grow, this approach may also offer a more effective use of healthcare resources within local systems, though further health economic evaluations are needed to fully assess their impact.

Finally, although current understandings of clinical demand for late effect services should not inhibit a policy response, there needs to be a better understanding from patients on the of the short- and long-term impacts of radiotherapy which should come from national and local collection of PRD and patient feedback.
